# Supporting teachers to actively respond to bullying and to build positive relationships with their students: effects of the T-SUPPORT training

**DOI:** 10.3389/fpsyg.2023.1236262

**Published:** 2023-10-13

**Authors:** Chloë Finet, Heidi Vandebosch, Anouck Lubon, Hilde Colpin

**Affiliations:** ^1^School Psychology and Development in Context, Faculty of Psychology and Educational Sciences, KU Leuven, Leuven, Belgium; ^2^Department of Communication Studies, Faculty of Social Sciences, University of Antwerp, Antwerp, Belgium

**Keywords:** bullying, teachers’ responses to bullying, teacher-student relationships, intervention, elementary education

## Abstract

Despite the central role that teachers can have in preventing and reducing bullying, they often feel insecure about how to deal with bullying. This study evaluated a short teacher training – called the Teachers SUPporting POsitive RelaTionships (T-SUPPORT) training – that aims to reduce bullying by supporting teachers in building positive teacher-student relationships and in actively dealing with bullying. The aim of the current study was to investigate whether the T-SUPPORT training resulted in higher quality teacher-student relationships, and more active and less passive responses to bullying incidents, and whether these improvements in turn resulted in lower levels of bullying victimization. In a Randomized Controlled Trial 10 Belgian primary schools were randomly assigned to an intervention or control condition. The Grades 4–6 teachers of the five schools in the intervention condition received the three-session school-based training; control teachers received no intervention. Grade 4–6 students (*N* = 964; 55 classrooms) in these schools completed questionnaires at pre- and post-test. In contrast to the hypotheses, results of the two-level linear mixed model analyses revealed no significant effect of the training on teacher-student relationship quality, teachers’ responses to bullying and bullying victimization. Yet, higher quality teacher-student relationships and more active teacher responses to bullying were significantly associated with less bullying victimization, whereas more passive responses were linked with more victimization. The latter findings are in line with theorizing and research on the role that teachers can play in reducing bullying.

## Introduction

Bullying – defined as repeated and intentional aggression directed towards others who have difficulty defending themselves because of a power imbalance (Olweus, 1993) – is a widespread problem in schools, affecting the lives of numerous children worldwide. Prevalence estimates obtained by a cross-national study of the WHO indicated that about 11% of the 11- to 13-year-old children reported to have been bullied at least twice a month in the past months (Inchley et al., 2020). These victims are at greater risk for various negative outcomes, regardless of the type of bullying (e.g., physical, verbal, or relational). Bullying has, for instance, been found to affect children’s socio-emotional well-being and school outcomes on the short and long run (e.g., Moore et al., 2017; Arseneault, 2018). Moreover, not only victims, but also children who are involved in bullying as perpetrators (e.g., Kelly et al., 2015) or bystanders (e.g., Midgett and Doumas, 2019) are at risk to suffer poor outcomes. Given the high prevalence of bullying and its long-lasting consequences, prevention and intervention against bullying are of utmost importance (Flannery et al., 2016).

Referring to the socio-ecological paradigm (Bronfenbrenner, 1977), bullying and victimization are influenced by a complex interplay of individual and contextual factors (Espelage, 2014; Menesini, 2019), which provides targets for prevention and intervention efforts. One important school-level contextual influence relates to teachers’ impact on the classroom peer ecology (Yoon and Bauman, 2014). More specifically, according to the classroom peer ecology model of Gest and Rodkin (2011) teachers can impact bullying dynamics in a more direct and a more indirect way. First, teachers can impact bullying directly through the strategies that they use to deliberately influence the peer relationships in their class (i.e., peer network-oriented practices), which include their responses towards bullying. Moreover, teachers can indirectly influence bullying through the quality of their daily interactions with their students (Gest and Rodkin, 2011). Supporting this model, a gradually emerging body of evidence has shown that teachers’ responses towards bullying (e.g., Wachs et al., 2019), and the general quality of teacher-student interactions (Troop-Gordon and Kopp, 2011) are associated with bullying levels in their class. Despite this central role of teachers in tackling bullying, some teachers feel insecure regarding how to best intervene against bullying (Fischer and Bilz, 2019; Yoon et al., 2020), do not always respond to bullying incidents (Wachs et al., 2019), or experience difficulties in using positive relational skills (Weyns et al., 2017). Furthermore, students do not always perceive their teachers’ responses to bullying incidents as adequate, and sometimes even fear that their teachers’ responses might aggravate the situation (Bradshaw et al., 2007).

These findings highlight the need for training that supports teachers in preventing and reducing bullying in an adequate way. In the past years, a growing number of multicomponent, schoolwide antibullying programs have been developed, and generally proven effective with meta-analytic effect sizes in the small to moderate range (Gaffney et al., 2019). Despite this, schools sometimes experience the implementation of these multicomponent programs as burdensome and demanding, and the associated costs and resources might further impede their implementation (Bradshaw, 2015). Research into the effects of specific program components is needed to give insight into the most effective strategies to tackle bullying; in turn, focusing on these strategies may enhance feasibility of school bullying prevention (e.g., Tolmatcheff et al., 2022). The current study focuses on teacher training as a more specific strategy to prevent and reduce bullying. More specifically, the main goal was to develop and evaluate a low-threshold teacher training, called the Teachers SUPporting POstive RelaTionships (T-SUPPORT; Deboutte et al., 2021) training. Based on the classroom peer ecology model by Gest and Rodkin (2011), T-SUPPORT aims to reduce bullying and its negative consequences by supporting teachers in (1) promoting positive teacher-child relationships, and (2) actively dealing with bullying. The study is innovative, first, by testing teacher training as a specific intervention and not as part of a schoolwide program. Second, the training in this study adds to current teacher anti-bullying training by targeting teachers directly. In existing anti-bullying intervention, teachers are often considered as “intermediates” or “facilitators” who transmit the program to the students, e.g., by giving lessons, rather than as the targets of the intervention (Gaffney et al., 2021). Research about teacher anti-bullying training as a stand-alone intervention targeting teachers directly is very scarce. A notable exception is the Bully Busters program (Newman-Carlson and Horne, 2004), a training aimed at strengthening teachers’ effective intervention in bullying situations. A small-scaled quasi-experimental study found positive effects on teachers’ self-reported knowledge about bullying, their responses to bullying, self-efficacy in dealing with bullying, and on (reduced) school referrals of bullying (Newman-Carlson and Horne, 2004), suggesting that teacher training targeting teachers’ skills can be an effective strategy in tackling bullying. Research regarding the effects of teacher training on student perceptions is lacking, to the best of our knowledge. Last but not least, the T-SUPPORT training includes a focus on positive relationships whereas most existing teacher training, including Bully Busters, focuses on preventing and reducing negative student behavior and interactions, thereby missing the potential benefit of fostering positive teacher-student relationships. Referring to Gest and Rodkin (2011), promoting positive teacher-student relationships can be an additional approach to prevent bullying. Such an approach can be considered more feasible and desirable for teachers, because interacting with students is part of their job and, as such, the intervention can be integrated in their daily classroom routine.

### Teacher-student relationships

As described above, the peer ecology model of Gest and Rodkin (2011) assumes that teachers can indirectly influence peer relationships in their class – including bullying dynamics – through the quality of their interactions with their students. Through these daily interactions between teachers and their students, teacher-student relationships evolve (Pianta et al., 2003). One theoretical framework for understanding the importance of teacher-student relationships comes from attachment theory (Bowlby, 1969). According to this theory, early experiences between children and their primary caregivers (e.g., parents) are of vital importance for children’s future development, because these experiences lead to the formation of internal working models, which contain expectations about the availability of others in times of need and which influence later interpersonal behavior and interactions (Bosmans and Kerns, 2015; Waters and Roisman, 2019). Although teachers are not primary caregivers and teacher-student relationships are not exclusive and are limited in time, an increasing body of research suggests that teachers can serve as temporary attachment figures for their students and that teacher-student relationships can be considered as *ad hoc* attachment relationships (Verschueren and Koomen, 2012). From this theoretical framework, the quality of teacher-student relationships is typically conceptualized along positive (e.g., closeness and emotional support) and negative (e.g., conflict) dimensions. Children who have more positive relationships (e.g., closer, less conflictual relationships) with their teachers, are thought to be able to use their teacher as a secure base to explore their classroom environment and safe haven to turn to in case of problems (Verschueren and Koomen, 2012). Children for whom the teacher is a secure base and a safe haven, are more likely to develop the emotion regulation skills and social competences needed to build positive relationships and, hence, be less vulnerable to victimization (ten Bokkel et al., 2023). Further, it can be expected that these children will be more likely to seek support from their teacher when being victimized, and that teachers might be better attuned to the needs of these students. This might in turn increase the teacher’s opportunities for detecting and intervening in the bullying (Reavis et al., 2010). Finally, it has been argued that students who have a good relationship with their teacher are less likely to be victimized, because bullies might be aware of this good relationship. As a consequence, bullies might estimate the risk of retaliation by the teacher as higher when they would bully students who have a good relationship with their teacher than when they would bully students who have a less positive relationship with their teacher (Elledge et al., 2016).

In line with these theoretical predictions, a growing body of research has demonstrated that teacher-student relationships can play a role in peer processes in general (Endedijk et al., 2022), and bullying victimization in particular (Troop-Gordon and Kopp, 2011; Demol et al., 2020). A meta-analysis by ten Bokkel et al. (2023) on the link between teacher-student relationship quality and bullying victimization revealed that dyadic and classroom-level teacher-student relationship quality was significantly associated with victimization, both cross-sectionally and longitudinally. Importantly, intervention research in other areas than bullying research has shown that teacher-student relationship quality is amenable to change through teacher training, and that improved teacher-student relationship quality in turn relates to better child outcomes (Kincade et al., 2020; Spilt and Koomen, 2022). Based on the discussed literature, one of the main objectives of the T-SUPPORT training was to reduce levels of bullying victimization (hereafter referred to as victimization) by supporting teachers in building positive relationships with their students.

### Teachers’ responses to bullying

In addition to indirectly influencing bullying and victimization through the relationships with their students, teachers may also impact bullying processes more directly through their deliberate peer-network oriented practices including their responses to bullying incidents (Gest and Rodkin, 2011). Teachers can respond to bullying in a variety of ways, and several conceptualizations exist to distinguish between these different types of teachers’ responses to bullying (for an overview, see Colpin et al., 2021). Although no consensus exists yet on which conceptualization is best, many of them distinguish between active and passive responses. Whereas active responses include strategies such as disciplining the perpetrator, supporting the victim, and contacting parents, passive responses encompass strategies such as advising victims to stand up for themselves or even not responding at all (Troop-Gordon and Quenette, 2010; Campaert et al., 2017). At present, research on the link between teachers’ responses and bullying is inconclusive as to which responses are most effective for reducing bullying. Nonetheless, active strategies for dealing with bullying have generally been found to be associated with lower levels of bullying, and passive strategies with higher levels (e.g., Campaert et al., 2017; Wachs et al., 2019; van Gils et al., 2022). One potential explanation is that active responses provoke or strengthen student beliefs that their teacher does not tolerate bullying. On the other hand, passive responses might elicit the belief that teachers do not care or blame the victim (Troop-Gordon and Quenette, 2010) which may, in turn, reinforce bullying behavior and reduce the likelihood that victims disclose the bullying to their teacher.

Despite the role that teachers’ active responses might play in counteracting bullying, teachers do not always actively intervene (Wachs et al., 2019). This lack of active responding might have to do with various reasons, including for instance experienced difficulties with identifying bullying situations, with assessing the seriousness of bullying incidents, and with responding to persistent bullying in an adequate way (van Verseveld et al., 2020). These findings clearly show the importance of training teachers in dealing with bullying. Evidence that teachers can benefit from such training comes from the meta-analysis of Van Verseveld et al. (2019) on teacher-level effects of antibullying interventions. This meta-analysis revealed that antibullying interventions have a positive, though small, effect on teachers’ responses to bullying. Building on this, the second main objective of the T-SUPPORT training was to train teachers to respond to bullying incidents in an active way, which we expected to predict lower levels of victimization.

## Hypotheses

The aim of the current study was to test intervention effects of the newly developed T-SUPPORT training. Building on the literature on malleability of teacher-student relationships (Kincade et al., 2020) and of teachers’ responses to bullying (van Verseveld et al., 2020), we hypothesized that the training would result in better teacher-student relationship quality and in more frequent use of active and less frequent use of passive responses to bullying. Moreover, in line with the model of Gest and Rodkin (2011), with attachment theory (Pianta et al., 2003), and previous research on the associations between teacher-student relationship quality and teachers’ responses to bullying on the one hand and victimization on the other (e.g., van Gils et al., 2022; ten Bokkel et al., 2023), we expected that the hypothesized improvements in teacher-student relationship quality and in teachers’ responses to bullying would be associated with lower levels of victimization. Note that these hypotheses suggest that teacher-student relationship quality and teachers’ responses to bullying mediate the hypothesized association between the T-SUPPORT training and victimization.

## Materials and methods

### Participants and procedure

In the school year 2020–2021 (during the COVID-19 pandemic), 10 elementary schools from the region of Flanders in Belgium were recruited to participate in the T-SUPPORT study, which had an RCT design. Prior to recruitment, ethical study approval was obtained from the ethics committee of the KU Leuven (G-2020-1612). The schools were randomly assigned to either the intervention or education-as-usual condition using a matched pairs design (Imai et al., 2009) in which the schools were paired on their current anti-bullying practices, school size and students’ socioeconomic status. In the subsequent school year 2021–2022, the grades 4–6 teachers of the intervention schools received the T-SUPPORT training. Students from grades 4–6 (*N* = 1,305 students in 61 classes) were invited to fill out digital questionnaires at a pre-test [October 2021; Wave 1 (W1)] and post-test assessment [March/April 2022; Wave 2 (W2)]. In between the pre-test (W1) and the post-test (W2) assessment, between November 2021 and March 2022, the teachers in the intervention condition received the T-SUPPORT training; the teachers in the control condition received no training. The time between the end of the training and the W2 data collection was on average 33.8 school days (SD = 12.99).

Participation of the students in the study was conditional on provision of active parental informed consent, which was obtained for 1,091 students (83.6%). As in one of the schools (control condition) only limited completed consent forms were returned by the parents of the students in grades 5–6 and as the reminder that was sent to the parents did not improve the response rate, the school decided to withdraw participation of these classes (one grade 5 class and two grade 6 classes). Of the resulting 1,073 students, only the students who took part in both waves (*N* = 1,012, 94.3%) were included in the current analyses. Moreover, classrooms in which the students reported about a different teacher at W1 and W2 due to long-term absence of the teacher (*N* = 48 students in 3 classrooms) were excluded from the analyses, because intervention effects could not be evaluated for these students. This resulted in a final sample of 964 students (51.9% girls) across 55 classes (29 intervention, 26 control) of Grade 4 (32.7%), Grade 5 (33.4%) and Grade 6 (33.9%). Students in this sample did not differ from the students who were excluded from the analytic sample on age, gender or on any of the W1 study variables (all *p*-values non-significant). Mean age of the students in the analytic sample was 10 years and 5 months (SD = 11 months, range 8 years and 3 months – 12 years and 9 months) at W1. The majority of the students were born in Belgium (94.8%) and spoke Dutch at home (91.3%). The teachers (*N* = 55) about whom these students reported were mainly female (78.2%) and had an average age of 41.8 years (SD = 10.3, range 21.0–61.0). The majority of the teachers were born in Belgium (96.4%), had a professional (80.0%) or academic (9.1%) bachelor’s degree, worked fulltime (74.5%) and had on average 18.5 years of teaching experience (SD = 10.4, range 0.0–41.0).

At both waves, the digital student-report questionnaires were administered using the Qualtrics Offline Survey App on tablets.^1^ Data collection took place during school hours and lasted maximum two school hours per class (per wave). First, the examiner (postdoctoral researcher or graduate student) briefly introduced the study and read out loud a definition of bullying (based on Olweus, 1993). Students also received a paper version of this definition so that they could reread it during the data collection. Students were asked to provide their informed assent prior to filling out the questionnaire if they agreed to take part in the study.

### T-SUPPORT training

The T-SUPPORT training (Deboutte et al., 2021) is a new school-based teacher training that aims to reduce bullying by supporting teachers in building positive relationships with their students, and in dealing with bullying in an active way (*cf.* supra; Gest and Rodkin, 2011). T-SUPPORT aims to strengthen teachers in promoting positive teacher-student relationships and in dealing with bullying by targeting teachers’ cognitions and skills regarding these two training targets. The training was developed as a group training for upper elementary school teachers (grades 4–6) and consists of three half day sessions, which corresponds to the amount of time a year that Flemish elementary schools can spend on teachers’ professional training. In the intervention condition of this study, the group training was organized per school. In each of the schools, it was provided to the group of 4–6 Grade teachers; principals and internal school counsellors were welcome to attend. The first session of the training focused on providing important background information on bullying and the associated consequences, on understanding bullying and on identifying bullying. In the second session, special attention was given to the role that teachers can play in teacher-student and student–student relationships, and to the importance of collaboration among teachers for counteracting bullying (Kollerová et al., 2021). Moreover, teachers were asked to reflect on their own strengths and needs with respect to building positive teacher-student relationships and dealing with bullying. Finally, in the third session, teachers were provided with a variety of practical strategies that they can apply to deal with bullying incidents. Throughout the three sessions, several intervention methods were used by the trainer to promote teachers’ actions for building positive teacher-student relationships and for responding to bullying, including psychoeducation, role play, self-reflection and skill rehearsal. At the end of each session, the teachers reflected on how they would like to apply the content of the session in their daily classroom practice. In addition, at the start of the second and third session, the teachers were asked about their implementation experiences (e.g., “Which actions have you taken and what was the result?”). All training sessions were provided by an expert professional trainer between November 2021 and March 2022. In the original planning, there were three to four school weeks between session 1 and 2 and six to seven weeks between session 2 and 3 for all schools. Due to Covid-related reasons, session 2 had to be postponed with a few weeks in two schools and in one of these schools, session 3 was postponed accordingly. Because of the COVID-19 restrictions that were in place, not all sessions could be organized in the schools. More specifically, all second sessions and one of the final sessions had to take place online. Of the 29 teachers in the training condition, 21 (72%) teachers attended all three sessions, 7 (24%) attended two sessions and for one teacher information on intervention dosage was missing. Further, teachers were asked during the W2 data collection whether they had applied what they had learned in the training in their classroom. Eleven (37.9%) teachers reported that they had been able to implement what they had learned in their classroom, 7 (24.1%) reported that they had not yet been able to implement it in their classroom but that they planned to implement it in future, and the remaining 11 teachers (37.9%) indicated that they had not applied what they had learned in their classroom. A variety of reasons for non-implementation were reported by these teachers, including for instance good class climate, no bullying incidents, lack of practical tools provided in the training, and no time to implement the training because of the large number of students and teachers that were absent because of the pandemic.

### Measures

#### Teacher-student relationship quality

The validated Child Appraisal of Relationship with Teacher Scale (CARTS; Vervoort et al., 2015) was administered to the students at W1 and W2 to assess the affective quality of the teacher-student relationship. In the current study, the closeness (four items, e.g., “I like to be with my teacher.”) and conflict subscale (seven items, e.g., “I often quarrel with my teacher.”) were used. All items had to be rated on a 5-point scale ranging from 1 (*not true*) to 5 (*true*) and subscale scores were computed as the mean of the subscale items. Cronbach’s alphas at W1 and W2 were 0.82 and 0.86 for closeness, and 0.81 and 0.87 for conflict.

In addition, the teacher-student interaction quality subscale of the Dutch Climate Scale (Donkers and Vermulst, 2011) was used to assess students’ perceptions of the quality of the teacher-child relationships in the class. This subscale consists of 11 items (e.g., “This teacher is interested in the pupils.”) that had to be rated by the students on a 4-point scale ranging from 1 [*(almost) never*] to 4 [*(almost) always*]. The subscale score was computed as the mean of the 11 items. Previous research supported the psychometric properties of this subscale (e.g., Donkers and Vermulst, 2011; Demol et al., 2020). Cronbach’s alpha in the current sample was 0.85 at W1 and 0.89 at W2.

#### Teachers’ responses to bullying

To measure teachers’ responses to bullying, the students filled out the Dutch version (van Gils et al., 2022) of the Teachers’ Responses to Bullying Questionnaire (TRBQ; Campaert et al., 2017; Nappa et al., 2020) at W1 and W2. This 15-item questionnaire assesses students’ perceptions of one passive teacher response to bullying, namely non-intervention (e.g., “My teacher does nothing.”), and of four more active teacher responses to bullying, namely disciplinary sanctions (e.g., “My teacher takes measures against the bully/bullies.”), victim support (e.g., “My teacher comforts the victim.”), mediation (e.g., “My teacher helps the students involved to solve the bullying.”), and group discussion (e.g., “My teacher discusses bullying with the whole class.”). All subscales consist of three items scored on a 5-point scale ranging from 1 (*never*) to 5 (*always*). Subscale scores were computed as the average of the relevant items. Previous research has found support for the factor structure, validity and reliability of the TRBQ (Nappa et al., 2020), although the reliability of the non-intervention subscale was low in a Flemish sample (van Gils et al., 2022). Cronbach’s alphas at W1 and W2 were 0.50 and 0.59 for non-intervention, 0.63 and 0.70 for disciplinary methods, 0.72 and 0.77 for victim support, 0.71 and 0.77 for mediation, and 0.72 and 0.77 for group discussion.

#### Victimization

Victimization was evaluated with the victimization subscales of the validated Social Experiences Questionnaire-II (SEQ-II; Crick and Grotpeter, 1996). The SEQ-II consists of two victimization subscales of five items each, namely relational victimization (e.g., “How often are you left out on purpose when it is time to play or do an activity?”), and physical/verbal victimization (e.g., “How often are you being yelled at and called mean names?”). Items had to be scored on a 5-point scale ranging from 1 (*never*) to 5 (*always*). Previous research provided support for the psychometric properties of the SEQ-II (Crick and Grotpeter, 1996). Because our study focused on general victimization and because both subscales were highly correlated at both time points (W1: *r* = 0.70, *p* < 0.001; W2: *r* = 0.69, *p* < 0.001), a total victimization score was calculated by averaging all ten items. Cronbach’s alphas of the total victimization scale at W1 and W2 were 0.90 and 0.91, respectively.

### Data analyses

#### Statistical analyses

Hypotheses and analyses were pre-registered on OSF after the data collection but prior to the data analysis.^2^ Prior to the main analyses, descriptive statistics and correlations were computed for all study variables. In addition, given the nested nature of the data (with students nested in classrooms), intraclass correlation coefficients (ICCs) were calculated for all W2 study variables to estimate the proportion of variability in these variables at the classroom-level. These ICCs were computed by fitting a two-level random intercept null model for each variable (i.e., a model with only a fixed intercept and a random intercept at the classroom-level). Furthermore, to determine whether the randomization of the schools in the training and control condition was successful, both conditions were compared on all W1 (baseline) study variables and on the background variables age and gender. These preliminary analyses were conducted in SAS Enterprise Guide 8.1.

Next, to investigate our research question regarding the mediating role of (A) W2 teacher-student relationship quality (i.e., closeness, conflict, and teacher-student interaction quality), and (B) W2 teachers’ responses to bullying (i.e., non-intervention, disciplinary methods, victim support, mediation, and group discussion) in the hypothesized association between the T-SUPPORT training and W2 victimization, we conducted multilevel regression analyses with a random classroom-level intercept to take the clustering of students (level 1) within classrooms (level 2) into account. Note that our model corresponds to a 2-1-1 mediation model, because the predictor (intervention) was assessed at level 2, and the eight hypothesized mediator variables (three indicators of teacher-student relationship quality and five teacher responses to bullying) and the outcome variable (victimization) were measured at level 1. The multilevel analyses were performed in SAS Enterprise Guide 8.1 using the PROC MIXED procedure with Restricted Maximum Likelihood Estimation (REML). All analyses controlled for W1 age and gender.

**Figure 1:**
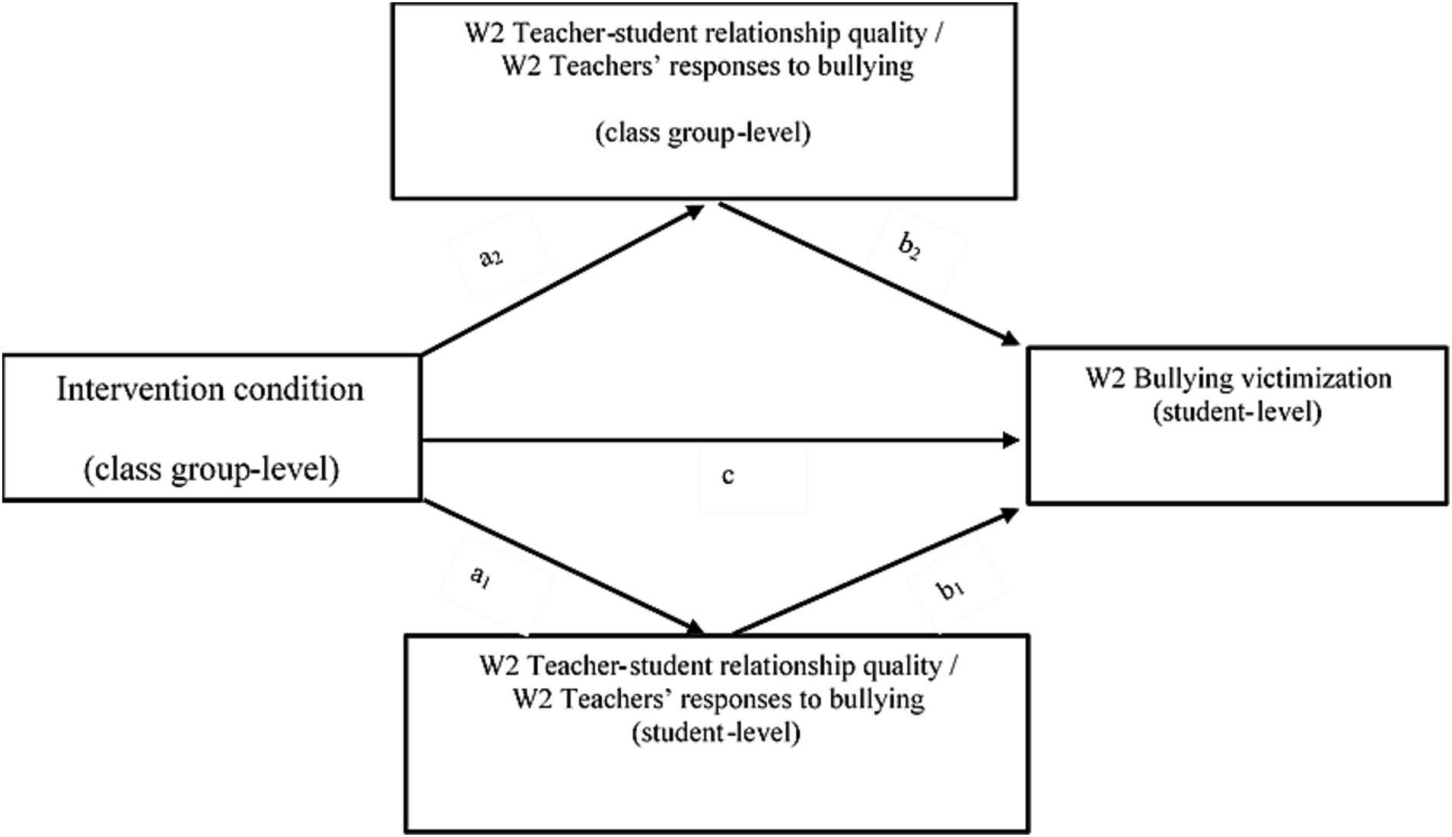
Direct effects of the intervention on W2 levels of victimization and indirect effects via the student-level mediators and via the class group-level mediators.

We adopted the cross-level mediation approach of Pituch and Stapleton (2012), because this approach allows investigating individual-level indirect effects (i.e., the indirect effect that flows through the individual level mediator; hereafter referred to as the student-level indirect effect) as well as cluster-level indirect effects (i.e., the indirect effect that flows through the cluster mean of the individual-level mediator; hereafter referred to as the class group-level indirect effect) in 2-1-1 mediation models. Hence, by adopting this approach (Pituch and Stapleton, 2012), both the within-class associations between the hypothesized mediators and victimization and the between-class associations can be estimated (Talloen et al., 2016). A graphical representation of the hypothesized mediation models is displayed in Figure 1. Separate mediation analyses were performed for each of the eight hypothesized mediators. The cross-level mediation approach of Pituch and Stapleton (2012) consists of three steps. Referring to Pituch and Stapleton’s (2012) three-step approach, in the first step, we fitted a two-level random intercept model in which we regressed W2 victimization on the intervention and on the covariates (W1 victimization, age and gender) to estimate the total effect of the intervention on victimization (c-path coefficient; Figure 1). This step is the same for all mediators and was, hence, only performed once.

In the second step, the effect of the intervention on the student-level mediators (a_1_-path coefficient; Figure 1) and on the class group-level mediators (a_2_-path coefficient; Figure 1) were estimated. More precisely, to investigate the effects of the intervention on the student-level mediators, we estimated two-level random intercept models with the W2 student-level mediator as dependent variable, the intervention as independent variable, and the W1 response on the corresponding mediator and age and gender as covariates. A separate model was fitted for each of the eight proposed mediators. To investigate the effects of the intervention on the class group-level mediator (i.e., the class group aggregate of the student-level mediators), single-level linear regression models were fitted in which the class group-level mediator was included as dependent variable, the intervention as independent variable, and the W1 response on the mediator and age and gender as covariates. To this end, class group-level aggregates of the predictor and dependent variables of these models were computed by calculating the class group average of the student-level responses per class group. Single-level linear regression models instead of two-level random-intercept models were fitted, because there is no within-classroom variation in the dependent variables of these models (i.e., variation between scores of students in the same class) due to the classroom-level aggregation of the student-level scores. For each of the eight class-group level mediators, a separate linear regression model was fitted. In the third and final step, the effect of the student-level mediators (b_1_-path coefficient; Figure 1) and of the class group-level mediators (b_2_-path coefficient; Figure 1) were estimated simultaneously by regressing W2 victimization on the student-level mediator and its class group-level aggregate, on the intervention, and on the covariates (W1 victimization, W1 response on the corresponding mediator, and age and gender). For each of the eight mediators, a two-level random intercept model was fitted.

Finally, if the conditions for mediation (for a particular mediator) were met, the student-level indirect effect was computed as the product of the estimated a_1_ and b_1_ coefficients and the class group-level indirect effect was computed as the product of the estimated a_2_ and b_2_ coefficients, and significance of both indirect effects was tested using the Monte Carlo Method for Assessing Mediation (MCAM; Selig and Preacher, 2008; Preacher and Selig, 2012). The MCAM computes the empirical sampling distribution of an indirect effect and uses this empirical sampling distribution to construct a 95% Monte Carlo confidence interval for the indirect effect. We used the interactive MCAM tool for R, provided by Selig and Preacher (2008), to construct the 95% confidence intervals for the indirect effects. The student-level and class group-level indirect effects were considered significant if their confidence interval excludes zero.

Prior to the main analyses, we examined the data for extreme outliers (defined as ±3.29 standard deviations away from the mean) in IBM SPSS Statistics (Version 27.0). The number of extreme outliers ranged between 0 and 15 (1.6%). All main analyses were repeated excluding these outliers. As excluding the outliers did not alter the results in terms of significance and direction of effects, we only report the results of the analyses in which the outliers were retained (Kutner et al., 2005).

In addition to the preregistered analyses, we calculated the correlations between the teacher characteristics age and gender on the one hand, and the W2 outcome variable victimization and all mediating variables aggregated at class level on the other. As all those correlations were non-significant, we concluded that it was not necessary to run additional analyses with the teachers’ age and gender as covariates. Moreover, as an additional exploratory analysis, the first and the second step, testing the direct effects of the intervention on the outcome variable victimization and on the mediator variables respectively, were conducted in a reduced sample, including the students in the control condition (*n* = 419) and only the students from the intervention condition whose teacher reported having practiced the training content in their classroom (*n* = 215). None of the tested effects differed from the analyses in the whole sample in terms of significance. Hence, only the results of the total sample are reported.

#### Missing data-analysis

The scale scores of all questionnaires were computed using listwise deletion at the item level, meaning that the scale scores (mean scores) were only computed if none of the corresponding items were missing. Within the analytic sample, the proportion of missing values on these scale scores and age and gender was below 1%. Hence, missing data was not imputed, but handled using REML (*cf.* supra).

## Results

### Preliminary analyses

Descriptive statistics and classroom-level ICCs are shown in Table 1. The classroom-level ICCs of the W2 study variables ranged from 0.03 to 0.14, indicating that between 3 and 14% of the variance in these variables were explained by between-classroom differences. Two-level random intercept models with intervention as fixed effect were performed to test for potential differences between the training and control condition on the W1 study variables and the covariates (age and gender). Results revealed that there were no significant differences between both conditions on these variables (all *p*-values nonsignificant at the 0.05 level).

**Table 1:** Descriptive statistics and classroom-level ICCs for the study variables.

	Total				Control		Training	
	*N*	*M*	SD	ICC	*M*	SD	*M*	SD
W1 age	958	10.38	0.89	-	10.37	0.86	10.38	0.90
W1 closeness	958	4.10	0.78	0.09	4.09	0.76	4.11	0.79
W1 conflict	961	1.52	0.58	0.06	1.53	0.59	1.51	0.57
W1 teacher-student interaction quality	961	3.33	0.47	0.15	3.32	0.49	3.34	0.45
W1 non-intervention	962	2.02	0.75	0.05	2.06	0.78	1.99	0.72
W1 disciplinary methods	960	3.88	0.77	0.06	3.89	0.79	3.87	0.76
W1 victim support	962	4.09	0.78	0.08	4.04	0.81	4.13	0.76
W1 mediation	962	4.22	0.77	0.08	4.16	0.82	4.27	0.72
W1 group discussion	961	3.27	0.99	0.17	3.17	0.95	3.35	1.02
W1 victimization	962	1.90	0.70	0.04	1.88	0.69	1.91	0.70
W2 closeness	955	4.05	0.86	0.10	4.07	0.81	4.03	0.89
W2 conflict	957	1.58	0.69	0.04	1.59	0.72	1.57	0.67
W2 teacher-student interaction quality	960	3.26	0.53	0.14	3.28	0.52	3.25	0.54
W2 non-intervention	961	2.10	0.75	0.05	2.14	0.74	2.07	0.77
W2 disciplinary methods	958	3.84	0.82	0.03	3.87	0.81	3.81	0.82
W2 victim support	961	3.98	0.84	0.05	3.98	0.83	3.98	0.84
W2 mediation	960	4.08	0.82	0.05	4.08	0.80	4.08	0.85
W2 group discussion	958	3.35	0.99	0.07	3.34	0.99	3.36	0.99
W2 victimization	963	1.81	0.69	0.03	1.76	0.67	1.85	0.71

W1 = Wave 1, W2 = Wave 2.

As indicated in Table 2, age was significantly negatively correlated with W2 closeness and W2 teacher-student interaction quality and significantly positively correlated with W1 conflict. Furthermore, age was significantly positively correlated with some active student-reported teacher responses to bullying (namely W1 and W2 disciplinary methods, W1 mediation, and W1 group discussion) and negatively correlated with W2 victimization. Regarding the correlations with gender, girls reported significantly higher teacher-student relationship quality than boys at both waves, as indicated by lower conflict, and higher closeness and teacher-student interaction quality. Moreover, girls generally perceived their teachers as using more active strategies for dealing with bullying at W1 (i.e., disciplinary methods, victim support, and mediation) and to engage less in non-intervention at W2, and girls reported lower levels of victimization than boys at W1. Besides, all correlations between the main study variables were in the expected direction. The correlations between the corresponding W1 and W2 variables were all significant. Furthermore, higher perceived teacher-student relationship quality (higher closeness, lower conflict, higher teacher-student interaction quality) was associated with lower levels of victimization at both waves. Finally, higher levels of non-intervention for dealing with bullying were significantly positively correlated with more victimization at both waves, and higher use of active strategies for dealing with bullying were generally significantly associated with lower levels of victimization at both waves.

**Table 2:** Pearson correlation coefficients between the W1 and W2 study variables and covariates.

	1.	2.	3.	4.	5.	6.	7.	8.	9.	10.	11.	12.	13.	14	15.	16.	17.	18.	19.	20.
1. W1 Closeness	–																			
2. W2 Closeness	0.58^***^	–																		
3. W1 Conflict	−0.54^***^	−0.47^***^	–																	
4. W2 Conflict	−0.40^***^	−0.60^***^	0.65^***^	–																
5. W1 TS interaction quality	0.67^***^	0.45^***^	−0.43^***^	−0.32^***^	–															
6. W2 TS interaction quality	0.50^***^	0.73^***^	−0.36^***^	−0.44^***^	0.60^***^	–														
7. W1 Non-intervention	−0.33^***^	−0.23^***^	0.25^***^	0.24^***^	−0.34^***^	−0.29^***^	–													
8. W2 Non-intervention	−0.22^***^	−0.42^***^	0.23^***^	0.30^***^	−0.25^***^	−0.46^***^	0.30^***^	–												
9. W1 Disciplinary methods	0.31^***^	0.19^***^	−0.13^***^	−0.13^***^	0.32^***^	0.22^***^	−0.28^***^	−0.18^***^	–											
10. W2 Disciplinary methods	0.24^***^	0.35^***^	−0.16^***^	−0.18^***^	0.21^***^	0.34^***^	−0.17^***^	−0.34^***^	0.38^***^	–										
11. W1 Victim support	0.44^***^	0.27^***^	−0.26^***^	−0.18^***^	0.46^***^	0.34^***^	−0.35^***^	−0.22^***^	0.55^***^	0.27^***^	–									
12. W2 Victim support	0.31^***^	0.47^***^	−0.21^***^	−0.25^***^	0.32^***^	0.50^***^	−0.29^***^	−0.44^***^	0.30^***^	0.63^***^	0.39^***^	–								
13. W1 Mediation	0.43^***^	0.26^***^	−0.28^***^	−0.22^***^	0.46^***^	0.31^***^	−0.37^***^	−0.24^***^	0.51^***^	0.25^***^	0.60^***^	0.31^***^	–							
14. W2 Mediation	0.30^***^	0.41^***^	−0.21^***^	−0.24^***^	0.29^***^	0.44^***^	−0.27^***^	−0.43^***^	0.23^***^	0.52^***^	0.30^***^	0.65^***^	0.35^***^	–						
15. W1 Group discussion	0.22^***^	0.13^***^	−0.01	0.01	0.25^***^	0.17^***^	−0.19^***^	−0.15^***^	0.45^***^	0.24^***^	0.45^***^	0.27^***^	0.37^***^	0.14^***^	–					
16. W2 Group discussion	0.13^***^	0.23^***^	−0.04	−0.03	0.14^***^	0.24^***^	−0.10^**^	−0.24^***^	0.25^***^	0.50^***^	0.24^***^	0.49^***^	0.19^***^	0.41^***^	0.46^***^	–				
17. W1 Victimization	−0.19^***^	−0.15^***^	0.29^***^	0.23^***^	−0.22^***^	−0.17^***^	0.18^***^	0.10^**^	−0.13^***^	−0.11^***^	−0.11^***^	−0.10^**^	−0.18^***^	−0.13^***^	−0.03	−0.01	–			
18. W2 Victimization	−0.12^***^	−0.19^***^	0.19^***^	0.27^***^	−0.13^***^	−0.21^***^	0.17^***^	0.19^***^	−0.12^***^	−0.15^***^	−0.07^*^	−0.16^***^	−0.14^***^	−19^***^	−0.04	−0.08^***^	0.65^***^	–		
19. Age	−0.06	−0.10^**^	0.10^**^	0.06	−0.04	−0.07^*^	0.04	0.03	0.08^*^	0.09^**^	−0.01	−0.06	0.07^*^	−0.03	0.18^***^	0.05	0.02	−0.07^*^	–	
20. Gender^a^	0.20^***^	0.22^***^	−0.24^***^	−0.25^***^	0.14^***^	0.11^**^	−0.10^**^	−0.09^**^	0.07^*^	0.05	0.10^***^	0.05	0.08^*^	−0.02	0.06	0.04	−0.06	−0.07^*^	−0.01	–

^a^ Gender with 0 = boy and 1 = girl. W1 = Wave 1, W2 = Wave 2. TS interaction quality = teacher-student interaction quality. * *p* < 0.05. ** *p* < 0.01. *** *p* < 0.001.

### Testing the direct and indirect effects of the T-SUPPORT training

Results of the analyses to test the mediating role of (A) W2 teacher-student relationship quality, and of (B) W2 teachers’ responses to bullying in the hypothesized association between the intervention and W2 victimization are displayed in Table 3 for the student-level indirect effects and in Table 4 for the classroom-level indirect effects. In the first step of the mediation analyses, results of the two-level random intercept model indicated that the intervention did not have a significant effect on W2 victimization, when controlling for W1 victimization, age and gender (see Table 3; c-path). In other words, the T-SUPPORT training did not have a significant effect on student victimization.

**Table 3:** Results of the two-level random intercept mediation models for estimating the student-level indirect effect of the intervention on W2 victimization via (A) W2 teacher-student relationship quality, and (B) W2 teacher responses to bullying.

				Fixed part			Random part					
							Class-level variance			Residual variance		
Predictor (level 2)	Mediator (level 1)	Outcome (level 1)	Path	*B*	SE	*p*	*B*	SE	*p*	*B*	SE	*p*
Intervention^a^		Victimization	c	0.07	0.04	0.097	0.01	0.005	0.037	0.27	0.01	<0.001
Intervention^a^	Closeness		a_1_	−0.07	0.07	0.317	0.03	0.01	0.003	0.44	0.02	<0.001
Intervention^a^	Conflict		a_1_	0.002	0.04	0.969	0.01	0.004	0.086	0.27	0.01	<0.001
Intervention^a^	TS interaction quality		a_1_	−0.04	0.04	0.306	0.01	0.004	0.003	0.17	0.01	<0.001
Intervention^a^	Non-intervention		a_1_	−0.04	0.06	0.518	0.02	0.01	0.013	0.49	0.02	<0.001
Intervention^a^	Disciplinary methods		a_1_	−0.04	0.05	0.395	0.001	0.01	0.459	0.57	0.03	<0.001
Intervention^a^	Victim support		a_1_	−0.05	0.06	0.408	0.01	0.01	0.068	0.57	0.03	<0.001
Intervention^a^	Mediation		a_1_	−0.04	0.06	0.470	0.01	0.01	0.176	0.59	0.03	<0.001
Intervention^a^	Group discussion		a_1_	−0.07	0.07	0.360	0.02	0.01	0.038	0.76	0.04	<0.001
	Closeness	Victimization	b_1_	−0.14	0.03	<0.001	0.01	0.004	0.066	0.26	0.01	<0.001
	Conflict	Victimization	b_1_	0.21	0.03	<0.001	0.01	0.004	0.042	0.25	0.01	<0.001
	TS interaction quality	Victimization	b_1_	−0.26	0.04	<0.001	0.01	0.004	0.049	0.26	0.01	<0.001
	Non-intervention	Victimization	b_1_	0.11	0.02	<0.001	0.01	0.005	0.034	0.26	0.01	<0.001
	Disciplinary methods	Victimization	b_1_	−0.06	0.02	0.014	0.01	0.004	0.060	0.26	0.01	<0.001
	Victim support	Victimization	b_1_	−0.09	0.02	<0.001	0.01	0.005	0.049	0.26	0.01	<0.001
	Mediation	Victimization	b_1_	−0.10	0.02	<0.001	0.01	0.005	0.042	0.26	0.01	<0.001
	Group discussion	Victimization	b_1_	−0.06	0.02	0.002	0.01	0.005	0.047	0.26	0.01	<0.001

^a^ Intervention with 0 = control condition and 1 = training condition. All mediators and outcome variables assessed at W2. TS interaction quality = teacher-student interaction quality. In the model for estimating the c-path (step 1) we controlled for W1 bullying, age and gender. In the models for estimating the a_1_-paths (step 2) we controlled for W1 score on the mediator, age and gender. In the models for estimating the b_1_-paths (step 3) we controlled for the W2 classroom-level aggregate of the mediator, W1 score on the mediating variable, W1 victimization, age and gender. All continuous predictors were grand mean centered.

**Table 4:** Results of the single-level linear regression analyses for estimating the effects of the intervention on the classroom-level mediators (A) W2 classroom-level teacher-student relationship quality, and (B) W2 classroom-level teacher responses to bullying (a_2_-paths) and of the two-level random intercept mediation models for estimating the effects of the W2 classroom-level mediators on victimization (b_2_-paths).

				Fixed part			Random part					
							Class-level variance			Residual variance		
Predictor (level 2)	Mediator (level 1)	Outcome (level 1)	Path	*B*	SE	*p*	*B*	SE	*p*	*B*	SE	*p*
Intervention^a^	Closeness		a_2_	−0.05	0.07	0.485	NA	NA	NA	NA	NA	NA
Intervention^a^	Conflict		a_2_	−0.001	0.04	0.980	NA	NA	NA	NA	NA	NA
Intervention^a^	TS interaction quality		a_2_	−0.03	0.04	0.443	NA	NA	NA	NA	NA	NA
Intervention^a^	Non-intervention		a_2_	−0.04	0.07	0.587	NA	NA	NA	NA	NA	NA
Intervention^a^	Disciplinary methods		a_2_	−0.02	0.05	0.755	NA	NA	NA	NA	NA	NA
Intervention^a^	Victim support		a_2_	−0.03	0.06	0.582	NA	NA	NA	NA	NA	NA
Intervention^a^	Mediation		a_2_	−0.04	0.05	0.399	NA	NA	NA	NA	NA	NA
Intervention^a^	Group discussion		a_2_	−0.05	0.07	0.473	NA	NA	NA	NA	NA	NA
	Closeness	Victimization	b_2_	0.10	0.06	0.111	0.01	0.004	0.066	0.26	0.01	<0.001
	Conflict	Victimization	b_2_	−0.16	0.10	0.126	0.01	0.004	0.042	0.25	0.01	<0.001
	TS interaction quality	Victimization	b_2_	0.18	0.10	0.059	0.01	0.004	0.049	0.26	0.01	<0.001
	Non-intervention	Victimization	b_2_	−0.02	0.09	0.840	0.01	0.005	0.034	0.26	0.01	<0.001
	Disciplinary methods	Victimization	b_2_	−0.07	0.09	0.424	0.01	0.004	0.060	0.26	0.01	<0.001
	Victim support	Victimization	b_2_	0.02	0.08	0.844	0.01	0.005	0.049	0.26	0.01	<0.001
	Mediation	Victimization	b_2_	0.05	0.08	0.559	0.01	0.005	0.042	0.26	0.01	<0.001
	Group discussion	Victimization	b_2_	0.05	0.06	0.457	0.01	0.005	0.047	0.26	0.01	<0.001

^a^ Intervention with 0 = control condition and 1 = training condition. All mediators and outcome variables assessed at Wave 2. TS interaction quality = teacher-student interaction quality. NA, not applicable. In the single level models for estimating the a_2_-paths (step 2) we controlled for the classroom-level aggregate of the W1 score on the mediator, and for classroom-level aggregated age and gender. In the models for estimating the b_2_-paths (step 3) we controlled for the W2 student-level mediator, W1 score on the mediating variable, W1 bullying, age and gender. All continuous predictors were grand mean centered.

In the second step, the effects of the intervention on the W2 student-level mediators (a_1_-paths) and W2 classroom-level mediators (a_2_-paths) were examined. Results of the two-level random intercept analyses that were performed to investigate intervention effects on the W2 student-level mediators revealed that the intervention did not have a significant effect on any of the three indicators of teacher-student relationship quality, nor on the five different teachers’ responses for dealing with bullying (see Table 3; a_1_-paths). Similarly, results of the linear regression analyses that were carried out to test intervention effects on the classroom-level aggregated mediators indicated that the intervention did not have a significant effect on any of the proposed mediators (see Table 4; a_2_-paths). Hence, we did not find significant effects of the T-SUPPORT training on teacher-student relationship quality or teachers’ responses to bullying, neither at the individual student nor at the classroom level.

In the third step, associations between the W2 student-level and classroom-level mediators on the one hand and W2 victimization on the other hand were examined. In total, eight different two-level random intercept models were fitted. Results of the two-level random intercept models revealed that the W2 student-level mediators (the different indicators of teacher-student relationship quality and of teachers’ responses to bullying) were all significantly associated with W2 victimization (see Table 3; b_1_-paths). More specifically, and in line with expectations, higher levels of closeness, lower levels of conflict and higher teacher-student interaction quality as reported by individual students at W2 were all significantly associated with lower levels of W2 victimization. Furthermore, lower levels of non-intervention and higher use of active strategies for dealing with bullying (disciplinary methods, victim support, mediation, and group discussion) as perceived by individual students at W2 were associated with less W2 victimization. In contrast to the significant effects of the W2 student-level mediators, the effects of the W2 classroom-level mediators on W2 victimization were all non-significant (see Table 4; b_2_-paths), indicating that class-level perceptions of teacher-student relationships and of the teacher’s responses to bullying were not significantly related to student victimization.

Because the conditions for mediation were not met for any of the mediators (student-level mediators: all a_1_-paths non-significant; classroom-level mediators: all a_2_-paths and b_2_-paths non-significant), we did not calculate the student-level and classroom-level indirect effects and did not estimate their significance.

## Discussion

The main aim of the current RCT was to evaluate intervention effects of the T-SUPPORT training. Based on the peer ecology model of Gest and Rodkin (2011), this new teacher training (Deboutte et al., 2021) aims to reduce bullying by supporting elementary school teachers in building positive relationships with their students and in dealing with bullying in an active way. We hypothesized that the teacher training would lead to better quality teacher-student relationships and to more active and less passive teacher responses for dealing with bullying, and that these improvements in turn would lead to lower levels of victimization. Our findings, however, did not support this hypothesized mediation model. The training did not have a significant effect on the proposed mediators, nor on victimization. Despite this, higher teacher-student relationship quality, more active responses for dealing with bullying and less passive responses were associated with lower levels of victimization. These significant associations were only found for between-student differences in teacher-student relationship quality and in perceived teachers’ responses, but not for between-classroom differences.

In contrast to our hypotheses, the T-SUPPORT training did not lead to a significant improvement in student-reported teacher-student relationship quality, nor to an increase in the use of active teacher strategies for dealing with bullying or a decrease in passive strategies. These findings stand in contrast with previous studies that showed that intervention programs are able to promote teachers’ strategies for improving teacher-student relationship quality (Kincade et al., 2020) and for dealing with bullying (van Verseveld et al., 2019), although not all programs were found to be effective and meta-analytic effect sizes appeared to be small to moderate. One possible explanation for the non-significant effects of the T-SUPPORT training relates to the fact that teachers did not get specific instructions regarding how to apply what they had learned in the training in their daily practice. Although they were invited to reflect upon how to apply the training skills in their classroom at the end of each session and to discuss how they applied the skills at the beginning of sessions two and three, they were not provided with manualized guidelines for their classroom behavior. This might have reduced implementation dosage and quality. Indeed, less than half of the teachers in the training condition indicated that they had been able to apply the training content in their classroom, although reasons for this low implementation rate also had to do with absence of (teacher-identified) bullying incidents. Although the current study was merely meant as a proof-of-concept, for future work it might be interesting to test whether the training can be improved by giving the teachers more manualized guidelines regarding how to implement the acquired skills and knowledge in daily practice. Second, the content and methods of the training might not yet be fully-suited to significantly impact the teacher-student relationship quality and teachers’ responses to bullying. Future research could extend the training with alternative evidence-based approaches to promote teacher-student relationships (for overviews, see Kincade et al., 2020; Poling et al., 2022), such as uncovering shared interests between teachers and students (Gehlbach et al., 2016) and teacher reflection on their mental representations of the relationship with their students (Spilt and Koomen, 2022). Regarding teachers’ responses to bullying, based on a recent study, Wolgast et al. (2022) argue that teacher training can be improved by enhancing teachers’ cognitive empathy, their awareness of violence and by teaching them interventions to effectively intervene in relational bullying situations. These three components were targeted in the T-SUPPORT training, but only to a limited extent, and could be more (explicitly) elaborated in future research. Third, the fact that the training only consisted of three sessions might provide an alternative explanation for the lack of intervention effects, as it has been shown that longer anti-bullying programs are generally more effective than shorter programs (Ttofi and Farrington, 2011). In addition to these training-related explanations, the COVID-19 pandemic and associated restrictions might have had an impact on the training. More specifically, some teachers were absent during the training due to illness or quarantine. In addition, due to the stricter restrictions that were imposed as response to the rise of the Omicron variant in the winter of 2021, the second training session had to be organized online, reducing the opportunity for active interactions and role play. This might have had a negative impact on the intervention, although no firm conclusions can be drawn about the impact that the pandemic had on the training’s efficacy. Finally, it might have been more challenging to detect potential intervention effects because the study variables were measured using student report, whereas not the students themselves but their teachers were the recipients of the training. In this respect it is noteworthy that Kincade et al. (2020) – who found that universal intervention programs can effectively improve teacher-student relationship quality – pointed out that all studies in their meta-analysis solely relied on teacher report. Hence, it is possible that the T-SUPPORT training might have had an effect on (part of) the teachers’ actions for improving teacher-student relationship quality and for tackling bullying, but that the students had not noticed these changes yet. Nonetheless, because the ultimate goal of the T-SUPPORT training was to reduce bullying, we decided to focus on the students’ perspectives in the current analyses as changes in teachers’ actions are more likely to affect student bullying when they are experienced by the students themselves (van Gils et al., 2023).

Furthermore, the training was not successful at counteracting victimization, the ultimate target outcome of the intervention. This is no surprise given the fact that our training did not have significant effects on the two hypothesized mediators (teacher-student relationship quality and teachers’ responses to bullying) that were targeted by our training. Moreover, inspection of the descriptive statistics showed that our sample was characterized by low mean levels of victimization at baseline, therefore not leaving much room for improvement. We believe that an interesting area for future research may be to examine whether intervention effects are moderated by students’ baseline involvement in bullying or students’ baseline quality of the relationship with their teacher. It is possible that students who are involved in bullying as perpetrators or victims or students who do not have a good relationship with their teacher, are more susceptible for improved teachers’ efforts for promoting teacher-student relationships and dealing with bullying and benefit more from these efforts. This idea is supported by an intervention study of Duong et al. (2019), in which it was found that a teacher training for improving teacher-student relationship quality was more effective among students who had low quality relationship with their teacher before the start of the intervention than among students who had a good relationship with their teacher.

Despite the lack of intervention effects of the T-SUPPORT teacher training on victimization, our findings revealed that higher teacher-student relationship quality, more active and less passive teacher responses to bullying were significantly associated with lower levels of victimization. This confirms previous (longitudinal) research regarding the association between teacher-student relationships (ten Bokkel et al., 2023) and teachers’ responses to bullying on the one hand (Wachs et al., 2019; van Gils et al., 2022) and bullying or victimization on the other. Moreover, these findings provide support for the model of Gest and Rodkin (2011) that states that teachers play a role in bullying dynamics via their practices for promoting positive relationships, and via their responses to bullying incidents. Note however, that these significant associations were only found when teacher-student relationship quality and teachers’ responses to bullying were measured at the individual student-level, but not when they were aggregated at the classroom-level. This suggest that students’ personal perceptions of the relationship with their teacher and of their teacher’s responses to bullying are more strongly related to victimization than students’ collective perceptions of these teacher actions. A possible explanation is that students’ relationships with and perceptions of the same teacher may differ (e.g., Sabol and Pianta, 2012). Likewise, perceptions of the teacher’s responses to bullying may vary across students, which was demonstrated in a recent study in the same age group (van Gils et al., 2023).

Although we did not find first support for the efficacy of the T-SUPPORT training and for the hypothesized mediation model, we believe the current study contributes to the literature because there is a dearth of research on what the effective components of antibullying programs are and on causal mechanisms that explain the effects of antibullying programs (Saarento et al., 2015; Tolmatcheff et al., 2022). The current study isolated teacher training as a component of interest and investigated whether the hypothesized intervention effects on bullying flow through teacher-student relationship quality and teachers’ responses to bullying. Despite the null findings regarding the efficacy of the T-SUPPORT training on victimization and on the proposed mediators, it might still be worthwhile to continue research efforts towards examining how teachers can be strengthened in their efforts to counteract bullying. The significance of such research efforts is highlighted by the continued need for improving anti-bullying interventions (Tolmatcheff et al., 2022), and by evidence on the key role that teachers play in preventing and dealing with bullying (Yoon et al., 2020).

### Strengths and limitations

The results of our study should be interpreted in light of some strengths and limitations. A first strength relates to the large sample of students that took part in the study and to the relatively high response rate and low drop out, despite the COVID-19 pandemic. Furthermore, the T-SUPPORT training was delivered to the teachers by an expert trainer according to a standardized manual (Deboutte et al., 2021). Finally, the study adopted a pre/post RCT design to evaluate intervention effects of the teacher training. However, the RCT design was limited by the fact that one school withdrew its participation shortly after allocation to the intervention condition. The main reason for the school’s withdrawal is that the school had decided to also take part in another project that focusses on classroom relationships, and that the school did not consider it feasible to combine participation in this project with participation in the T-SUPPORT study. To reduce the impact of this school’s withdrawal, the school was replaced by another school that had indicated to be interested in participation in the study regardless of treatment allocation.

A second limitation of the study has to do with the time span between the third training session and W2 data collection. For practical reasons, this time span was relatively short (minimum three school weeks), limiting the opportunity to detect potential intervention effects, as effects on the included outcomes might have needed more time to unfold. Third, although development of the T-SUPPORT training was based on prior research on experienced teacher needs (e.g., van Verseveld et al., 2020) and feedback about the training was obtained from a small group of teachers before the actual start of the study, it can be considered a limitation that our training was not tailored to the specific needs of the participating schools. This might have hampered the teachers’ motivation and implementation of the training (Han and Weiss, 2005). Future intervention research might profit from designing tailored interventions that are customizable to the needs of each particular school. Furthermore, our study was limited by its unidirectional focus on how teachers impact bullying dynamics, whereas teachers and students are involved in complex bidirectional interactions (e.g., Demol et al., 2020) and are influenced by the broader school context (Spilt et al., 2022). Although we are aware of these complex dynamics, we adapted a unidirectional approach because we were specifically interested in examining whether the training impacts victimization through its hypothesized influence on the mediators. Finally, it is important to note that the reliability of the non-intervention and disciplinary methods subscales of the TRBQ (van Gils et al., 2022) was rather low. Similar concerns regarding the reliability of some of the TRBQ subscales and other instruments for assessing teacher responses have been raised in previous research (van Gils et al., 2022), pointing to the need for novel instruments.

## Conclusion

The main aim of the current study was to develop and evaluate a new, short term teacher training – the T-SUPPORT training – that aims to reduce bullying by supporting teachers in promoting positive teacher-student relationships and in dealing with bullying. Contrary to expectations, the T-SUPPORT study did not have a significant effect on victimization, nor on the hypothesized mediators. Yet, higher quality teacher-student relationships, more active teacher responses to bullying and less passive responses were associated with lower levels of victimization. These results together with previous research showing that some teachers experience difficulties with responding to bullying in an adequate way (van Verseveld et al., 2020) and with promoting positive teacher-student relationships, demonstrate the need for continuous research efforts on how teachers can be supported in counteracting bullying and on causal mechanisms of intervention effects.

## Data availability statement

The raw data supporting the conclusions of this article will be made available by the authors, without undue reservation.

## Ethics statement

The studies involving human participants were reviewed and approved by Social and Societal Ethics Committee (SMEC) of the KU Leuven. Written informed consent to participate in this study was provided by the participants and the participants' legal guardian/next of kin.

## Author contributions

CF contributed to the design of the study, data collection, data analysis and interpretation, and drafted the manuscript. HV contributed to the funding acquisition, conception and design of the study, and participated in drafting the manuscript. AL contributed to the data collection and drafting of the manuscript. HC contributed to the funding acquisition, conception and design of the study, data analysis and interpretation, and participated in drafting the manuscript. All authors contributed to the article and approved the submitted version.

## Footnotes

^1^ https://www.qualtrics.com

^2^ https://osf.io/zg6n2
